# Theory of Mind in Adolescents with Bipolar Disorder in Euthymic Phase: ‎Using the Strange Stories Test

**Published:** 2016-07

**Authors:** Hamid Zarrabipoor, Mehdi Tehrani-Doost, Zahra Shahrivar

**Affiliations:** 1 Department of Psychiatry, Tehran University of Medical Sciences, ‎Roozbeh Hospital, Tehran, Iran.; 2 Research Center for Cognitive and Behavioral Sciences, Tehran University of Medical Sciences, Tehran, Iran.

**Keywords:** *Adolescents*, *Bipolar Disorder*, *Social Cognition*, *Theory of Mind*

## Abstract

**Objective: **This study evaluated the theory of mind (ToM) in adolescents diagnosed with bipolar disorder ‎‎(BD) during their euthymic period compared to a typically developing (TD) group.‎

**Method:** The BD group consisted of thirty 11-18 year old inpatients in euthymic phase. The TD ‎group included 30 age, gender, and IQ matched volunteer students. To assess the diagnosis and ‎comorbid disorders, we performed the semi-structured interview of the Kiddie Schedule for Affective Disorders ‎and Schizophrenia-Present and Lifetime Version (K-SADS-PL) for the BD adolescents. To ‎evaluate the severity of attention deficit hyperactivity disorder (ADHD) and mania, Conner's ‎Parent Rating Scale-Revised version (CPRS-R), and Young Mania Rating Scale (YMRS) were ‎used, respectively. Ravens Progressive Matrices was conducted to evaluate intellectual ability in ‎the both groups. Happe Strange Stories test was performed to assess ToM in the participants. Data were ‎analyzed using the independent t-test, analysis of covariance, and Pearson Correlation analysis.‎

**Results:** The two groups did not show any differences in comprehending the stories; however, the BD ‎group’s mentalizing scores were significantly weaker than the TD group (p<0.05).‎‎

**Conclusion**: The ToM impairments in adolescents with BD may be explained as a trait marker which may lead ‎to continuation of social problems even during remission‏.‏

Bipolar disorder (BD) has been described with periods of mania, depression and remission. The ‎lifetime prevalence of BD type I has been reported to be 1% in adolescents (1, 2). However, ‎adolescents with BD type II form 10% of the society and this disorder mostly occur between ‎the ages of 14 and 18 years (3) ‎‏.‏

Evidence shows that 20-50% of the patients with BD have chronic social impairment (4)‎‏ ‏and ‎difficulty in interaction with others (5) as well as a reduction in social relationships and ‎dissatisfaction in family and social interactions (6). Deficits in social relationships in these patients ‎continue even during the period of remission (4); however, the existing findings are inconsistent ‎‎ (7).‎

To understand the underlying mechanisms of social functioning, social cognition has been ‎explored as an advanced mental process essential to understand other’s intentions and attitudes ‎‎ (8). Social cognition includes subcategories such as relationship dynamics, social knowledge and ‎perception (9).‎

Research on social cognition has recently focused on theory of mind (ToM). ToM is a term used ‎to describe such mental states conceptualizations as beliefs, desires, intentions and emotions (10). ‎ToM also refers to a process through which people predict and interpret others’ behaviors (11). ToM related deficits in social ‎interactions have been examined repeatedly in autism and schizophrenia spectrum disorders (12), ‎and recently in individuals with BD (13). Kerr et al. (2003) (14) compared the ability of ToM ‎between BD adult individuals in normal, depressive, and manic phases, and a healthy group. First ‎and second false-belief tasks showed that ToM was impaired in the periods of depression and ‎mania, but was intact in euthymic phase. Montag et al. (2010) (15) and Olley et al. (2005) (16) did ‎not find any ToM impairment in the period of normal mood. However, Bora (2005) (17) showed ‎that ToM was weak in people whose mood disorder had been treated. Wollf (2010) (18) found ‎that ToM was more impaired in all BD phases compared to the healthy group. A recent meta-‎analysis (19) confirmed a significant modest ToM dysfunction in subclinical and remitted BD, and ‎a higher ToM dysfunction in acute episodes. These deficits may have an important role in ‎interpersonal problems seen in individuals with BD. ‎

Moreover, some studies have found social dysfunction in pediatric BD (20, 21 and 22). Besides, study ‎of offspring of parents with BD found social impairment in these youths (23). Whitney et al. ‎‎ (2014) (24) suggested that children and adolescents may show social impairment before they ‎experience the onset of mania, a fact consistent with common chronic prodromal period before ‎the emergence of pediatric BD (25). Schenkel et al. (2008) (26) conducted the first study on ToM in ‎pediatric BD and found that ToM functions of the adolescents with BD during the periods of ‎depression and mania were weaker than the healthy group. Moreover, Schenkel et al. (2014) (27) ‎showed that poor interpersonal functioning in pediatric BD type I was associated with ToM ‎deficits. They did not find any significant differences between pediatric BD type II and healthy ‎group in terms of mentalizing ability.‎

The ToM literature on euthymic phase of BD in adolescents is still scant and inconsistent. ‎Moreover, most studies have used parents reports or / and first or second order false belief tasks to ‎test ToM abilities (first order means to infer someone’s mental state; second order means to infer ‎someone mentalizing about another person’s mental state). However, advanced instruments are ‎available to evaluate higher levels of mentalizing process. For example, the strange stories test ‎assesses individuals’ understanding of pretending, double bluffing, lying, persuading, etc. In ‎addition, the studies on ToM in Iranian children and adolescents with BD are limited. These ‎facts encouraged us to study the underlying mechanisms of impairment in the social relationships ‎of adolescents with BD when they are in euthymic phase. We hypothesized that the ability of the ‎adolescents with BD in understanding others’ higher mental states when they are in the remission ‎phase is lower than the healthy group.‎

## Materials and Method

***Participants***

The study population consisted of 11-18 year old adolescents with BD (n = 30) and their normal ‎developing counterparts (n = 30). The clinical group was recruited from inpatients in the child ‎and adolescent ward at Roozbeh hospital. The typically developing individuals were volunteers ‎from mainstream schools. Exclusion criteria for both groups included any neurological or ‎developmental disorder, any history of head trauma, alcohol consumption, any physical illness ‎influencing cognitive functioning, and IQ lower than 90.‎

After explaining the study and obtaining informed consent from the participants (written ‎permission from parents and verbal permission from adolescents), we included those adolescents ‎who had been diagnosed with BD in acute, manic or mixed period by a child and adolescent ‎psychiatrist based on DSM IV criteria. The primary evaluations were as follows:‎

‎1.‎ To verify the diagnosis and examine comorbid disorders, a skilled psychologist ‎conducted the semi-structured interview of the Kiddie-Schedule for Affective ‎Disorders and Schizophrenia-Present and Lifetime version (K-SADS-PL).‎‏ ‏

‎2.‎ Young Mania Rating Scale (YMRS) was completed to determine the intensity of the ‎symptoms of mania in the BD group.‎

‎3.‎ With respect to the high prevalence of attention deficit-hyperactivity disorder ‎‎ (ADHD) in patients with BD, we used Conner’s Parent Rating Scale-Revised (CPRS-‎R) to evaluate the related symptoms.‎

‎4.‎ Raven's progressive matrices test was used to assess non-verbal intellectual ability.‎

‎5.‎ After 2-3 weeks, the YMRS was conducted again. If the score had been reduced to ‎eight or less, it was considered as an indicator of partial remission and patient’s ‎readiness to cooperate in doing ToM tests. Then, the participants were asked to attend the ‎neurocognitive lab at Roozbeh hospital to take the Happe Strange Stories test, which was conducted by a trained ‎psychologist.‎

The researchers did not intervene in the treatment process, but recorded the prescribed ‎medications. The healthy group was matched with the BD adolescents based on their age, gender ‎and intelligence. The Raven's progressive matrices, K- SADS-PL, CPRS-R, and Happe Strange Stories ‎test were also conducted in this group.‎

***Research Instruments***

*Happe Strange Stories Test:* This test (28) has been used as an advanced task of ToM assessment. It ‎consists of five groups of stories including mental states, human, animal, physical, and unlinked ‎sentences. The stories were displayed on a laptop screen while the participants were listening to ‎the narrator. Then they were asked to answer the questions about the stories. All answers were ‎recorded as well as written down by the examiner. Answers were scored “2” if the participants ‎had mentioned the main reason in the story process, and “1” if they pointed to it partially, and ‎‎“0” if their explanations were wrong or irrelevant. The Persian version of this test has been validated on ‎‎399 school-aged children in Tehran; the internal consistency (coefficient of Alpha Cronbach) was ‎‎.080 and the test-retest reliability was good (r = 0.713, p = 0.001) (29).‎

*Schedule for Affective Disorders and Schizophrenia for School-Age Children-Present and Lifetime* *‎* *Version (K-SADS-PL):* This is a semi-structured interview which evaluates the current and ‎lifetime psychiatric disorders and intensity of symptoms in children and adolescents (30). ‎Shahrivar et al. (2010) (31) found this instrument to have a good-to-excellent concurrent validity in ‎diagnosing current major disorders; its test-retest reliability in diagnosing ADHD and ODD was ‎also excellent.‎

*Young Mania Rating Scale (YMRS):* This instrument was used to evaluate the intensity of mania ‎symptoms. This instrument, scores 28 symptoms of mania on a 6-point Likert-type scale ranging ‎from 0-5. The internal consistency of this instrument was found to be good (32). Shafiee et al. (33) ‎found the concurrent validity of YMRS to be 0.87 and its validity and reliability to be acceptable ‎for research and clinical purposes.‎

*Conners' Parent Rating Scale-Revised-Short Form (CPRS-R: S):* This is a 27-item questionnaire ‎‎(34) which is completed by parents and produces oppositionality, inattention problems, ‎hyperactivity-impulsivity, and the ADHD indices. Tehrani-Doost et al. confirmed the validity of ‎this questionnaire to differentiate a healthy group from patients with ADHD (unpublished).‎

*Raven's Progressive Matrices (RPM):* This test includes a sequence of image patterns arranged ‎based on a special logic. Participants should complete each pattern by choosing a picture from ‎several pictures. Rahmani (1386) has found psychometric properties of this test in Iran (35) and found that it has good ‎validity and reliability.‎

***Statistical Analysis***

We performed the independent t test to compare the two groups in terms of the ToM variables ‎using the SPSS 16. Moreover, the regression analysis was conducted between the ToM variables ‎and ADHD symptoms. An analysis of covariance was performed to control the confounding ‎effect of CPRS hyperactivity score.‎

## Results

Demographic characteristics of the two groups are presented in Table 1.The two groups did not ‎have any significant differences in terms of age, gender and intelligence.‎ The average score of the YMRS for the BD group at the admission point was 26, and 6 in the ‎remission phase. The two main medications used in this group included lithium and sodium ‎valproate. In the BD group, 14 patients were diagnosed as having ADHD and one patient had ‎OCD. The CPRS-R: S hyperactivity subscale score was higher in the BD group (p = 0.01). ‎Considering the high comorbidity of ADHD and BD in the patient group, regression analysis ‎was conducted on ToM and CPRS-RS variables. If the results were significant, covariance ‎analysis was used, and if not, the independent t-test to compare the means (Table 2). ‎ Because the regression analysis revealed a significant relationship between the hyperactivity-‎impulsivity index and animal stories variable (t = -2.27, p<0.05), the covariance analysis was used ‎to control the effect of this index. According to the covariance analysis, the BD group’s scores ‎were lower than the TD group’s scores (p = 0.001).‎

‎, The average scores of mental states and nature stories were significantly higher in TD group ‎compared to the BD group (p = 0.001) (Table 3). Based on the two groups’ answers to the ‎unlinked sentences, there were no significant differences in terms of their comprehension. The ‎nature and physical stories assess the individuals’ understanding of causality related to animal’s ‎and human’s behaviors, while the mental states stories evaluate the ability of mentalizing

**Table1:** Demographic Characteristics of the Bipolar Disorder and Typically Developing Groups

	**Bipolar Group** **(n = 30)**	**Typically Developing Group** **(n = 30)**	**t**	**p**
Gender	Boys = 9 Girls = 21	Boys = 9 Girls = 21		
IQ (mean and SD)	107.3 (11.15)	111.8 (10.55)	0.09	-1.71
Age (year) (mean and SD)	15.7 (1.48)	15.4 (1.67)	0.09 0.57	-1.71 0.57

**Table2:** The Results of the Regression Analysis between the Strange Stories Test and CPRS-RS* Hyperactivity-Impulsivity Score

**Strange Stories**	**Unstandardized Coefficients**		**Standard Coefficients**	**t**	**Sig.**
	**B**	**Std. Error**	**Beta**		
Mental states	-0.351	1.051	-0.059	-0.334	0.740
Human	0.033	1.080	0.005	0.031	0.976
Animal	-2.087	0.919	-0.464	-2.271	0.027
Nature	1.294	0.974	0.243	1.329	0.189
Unlinked sentences	0.797	0.636	0.180	1.254	0.215

* CPRS-RS: Conners’ Parent Rating Scale-Revised Short Form

**Table3:** Comparison of Strange Stories Test Scores between the Two Group‎s

**Strange stories**	**Group**	**Number**	**Mean**	**Standard deviation**	**DF**	**T**	**Sig**
Physical	BD TD	30 30	10.66 11.08	2.73 1.76	58	-1.98	0.06
Mental states	BD TD	30 30	10.03 12.16	2.74 1.76	58	-3.63	0.001
Nature	BD TD	30 30	9.86 11.8	3.04 2.09	58	-2.86	0.001
Unlinked sentences	BD TD	30 30	8.63 10.36	3.71 2.31	58	-1.64	0.1

## Discussion

Considering the persistence of social problems in youths with BD during the euthymic period, we ‎compared the ability to understand others’ mental states in adolescents with manic or mixed BD and in healthy group using an advanced ToM task, the Happe Strange Stories test.‎

To our knowledge, our study was the first to examine ToM ability among adolescents with ‎BD during normal mood period. As hypothesized, our findings showed that ToM of the ‎participants with BD was weaker in the euthymic phase compared to the normal developing ‎group. ‎

Bipolar disorder mostly appears during adolescence, a period to begin establishing friendly and ‎intimate relationships with peer groups (3). Although the prominent symptoms of BD are ‎emotional, evidence shows that this disorder primarily results from cognitive dysfunction. ‎Consequently, problems in social relationships are caused by dysfunction in cognitive and ‎emotional circuits in the brain (36). In fact, emotional expressions of BD originate from an ‎impairment in cognitive control of these emotions (37). Moreover, it seems that the functional ‎deficiency of the neural systems involved in BD play a role in ToM. Neural circuits involved in ‎ToM include the cingulate cortex (monitoring the correct and incorrect selections), the medial ‎prefrontal cortex (the ability of self-reference), and the orbitofrontal cortex (evaluating and ‎processing emotional stimuli) (38). Therefore, considering the low capacity of monitoring others ‎and their intentions, the presence of psychotic symptoms such as control and persecutory ‎delusions, disorganized thought and speech, and some other behavioral symptoms in patients ‎with bipolar disorder are explainable (39).‎

Evidence is stronger for ToM impairment during the periods of elation and depression in BD (19). ‎Although some findings confirm difficulties in understanding other people’s mental states only in ‎the period of abnormal mood (14, 15 and 16), some authors indicated that this deficit continues even ‎into the period in which symptoms subside (17 and^18^). ‎

Different reasons have been suggested to explain ToM deficits in euthymic phase of BD (40). Of these explanations is that the persistence of ToM impairment into the euthymic period results from subliminal ‎mood symptoms which have not been cured completely, but the condition is not severe enough ‎to be diagnosed as an active period of mood disorder. On the other hand, socio-emotional ‎dysfunction may occur before the onset of mania in individuals with BD. Whitney et al. (2013) ‎‎ (41) compared a group of children and adolescents with a parent diagnosed with BD to ‎individuals who had no history of personal or family psychopathology and found numerous ‎social deficits in the high-risk group. They suggested that ToM and other social impairments in ‎the high-risk children might be due to the differences in innate brain functioning underlying ‎socio-emotional abilities or secondary to mood dysregulation affecting these youths’ normal ‎development.‎

Although some of the cognitive abilities (e.g., executive functions) decrease as age increases (42) ‎and this decrease occurs faster in BD, including attention and problem-solving (43), the increase of ‎age does not make ToM ability to decrease. In contrast, research indicates that age has a ‎determining role in predicting the ability of conceptualization (44). Using the Strange Stories test on ‎primary school children showed an age effect to predict ToM ability. Older children had higher ‎scores in mentalizing stories, and some abilities would not be observed before a higher age level ‏‎ (45). Therefore, any disruption in the development of social cognition including ToM may lead ‎to interpersonal problems. Emergence of BD during adolescence can impair this trajectory of ‎socio-emotional maturation.‎

## Conclusion

Bipolar disorder in adolescents is associated with social cognition problems which continue into ‎euthymic phase. Theory of mind deficit plays a great role in social impairment seen in youths ‎with remitted BD. Therefore, early evaluation and improvement of social cognition abilities in ‎adolescents with BD may be helpful in preventing their greater functional deterioration.‎ ‎

## Limitations

This study should be interpreted in light of some limitations. First, because of the ethical issues, ‎we continued prescribed medications, a factor which could have affected the participants’ ‎cognitive functioning. Second, ToM test was not performed during the acute mood episode, so ‎comparing ToM ability in acute and remitted periods was not possible. Third, co-occurrence of ‎ADHD with BD in our participants limited the generalizability of the findings, although it was ‎controlled as a confounding factor using the statistical methods. Fourth, the sample size was ‎rather low; therefore, the results should be considered with caution. Conducting longitudinal ‎studies to explore ToM during different phases of BD will be of great use. ‎
